# Acute Viral Hepatitis E Presenting With Bell’s Palsy and Acute Pancreatitis: A Case Report

**DOI:** 10.7759/cureus.73260

**Published:** 2024-11-07

**Authors:** Sayan Malakar, Sanjit Kumar, Sumit Rungta, Srikanth Kothalkar, Arvind Kumar, Krishna P Kohli, Rahul Jangra, Gaya P Shukla, Mayank Agarwal, Dheeraj Yadav, Anubhav Parwar, Saurabh Mishra, Akriti Bhardwaj

**Affiliations:** 1 Gastroenterology, King George's Medical University, Lucknow, IND

**Keywords:** acute pancreatitis, bell's palsy, cholestatic jaundice, extrahepatic manifestations, hepatitis e

## Abstract

Hepatitis E is a hepatotropic virus and the most common cause of acute viral hepatitis among adults in India. It has four genotypes, and genotype 1 is mostly associated with sporadic cases. It typically causes self-limiting acute hepatitis following a prodromal course. However, a subset of patients presents with cholestatic features mimicking primary cholestatic liver diseases like primary biliary cholangitis or primary sclerosing cholangitis. Liver injury ranges from asymptomatic rise of liver enzymes to fulminant liver failure. The hepatitis E virus (HEV) has also been implicated in various extrahepatic manifestations such as acute pancreatitis, Guillain-Barré syndrome (GBS), radiculopathy, autoimmune hemolysis, and Bell’s palsy. We present an interesting case of acute viral hepatitis E presenting with cholestatic jaundice and extrahepatic manifestations.

## Introduction

Hepatitis E is the most common cause of acute viral hepatitis in the adult population in India. It has four genotypes [1]. While genotypes 1 and 2 exclusively infect humans, genotypes 3 and 4 naturally circulate among other mammals. Genotype 1 is more prevalent in India and associated with sporadic outbreaks [1,2]. Jaundice in acute viral hepatitis E mostly resolves spontaneously. However, 5-10% of patients may have a prolonged or relapsed course of acute hepatitis [1,2]. Varied hepatic and extrahepatic manifestations have been described with acute viral hepatitis E [3]. Apart from hepatic injury, extrahepatic manifestations include pancreatitis, neurological manifestations, and autoimmune diseases [3,4]. Though rare, acute hepatitis E infection has been implicated as a cause of acute pancreatitis in India [5]. Extrahepatic neurological manifestations include aseptic meningitis, Guillain-Barré syndrome (GBS), peripheral neuropathy, and Bell’s palsy [6,7]. We present an interesting case of acute viral hepatitis complicated by Bell’s palsy and acute pancreatitis, which was successfully managed.

## Case presentation

**Figure 1:**
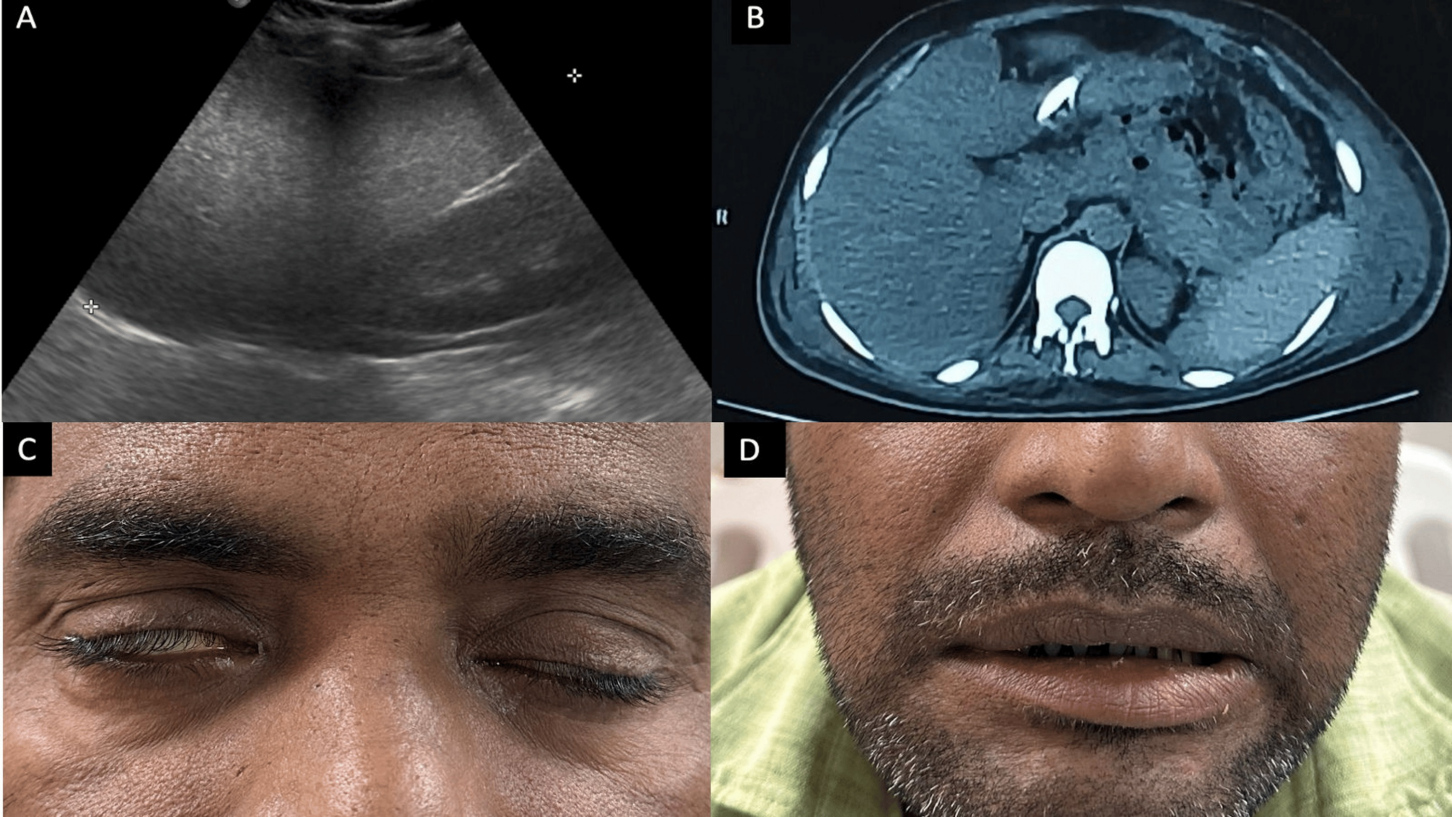
Extrahepatic manifestations of acute viral hepatitis E Figure A: Ultrasonography abdomen showing hepatomegaly. Figure B: CT of the abdomen showing bulky pancreas with peri-pancreatic fat standing suggestive of acute pancreatitis. Figure C: Figure showing Bell's phenomenon. Figure D: Facial asymmetry, depicting the features of tight-sided lower motor neuron type 7th cranial nerve palsy features CT: computed tomography

A 36-year-old male without any known comorbidity presented with complaints of jaundice and epigastric pain for 20 days. Before the onset of jaundice, the patient had a history of fever, joint pain, and malaise. There was no history of cough with sputum or burning micturition, any alcohol use disorder, smoking, or intravenous drug abuse. The patient had icterus, epigastric tenderness, and mild hepatomegaly. His lab examination results were as follows - total bilirubin: 8 mg/dl, direct bilirubin: 4 mg/dl, aspartate aminotransferase: 302 IU/L, alanine aminotransferase: 355 IU/L, alkaline phosphatase: 971 IU/L, serum amylase: 490 IU/L, and lipase: 356 IU/L. His ultrasonography report showed an enlarged liver (17 cm)(Figure 1A) and a normal portal vein and spleen. Given high persistent epigastric tenderness and high lipase, he underwent contrast-enhanced CT (CECT) abdomen. The pancreas was bulky and heterogenous with peri-pancreatic fat stranding (Figure 1B).

Based on history, clinical examination, and laboratory parameters, a diagnosis of acute hepatitis with acute pancreatitis was made. On further workup for acute hepatitis, IgM hepatitis E virus (HEV) was positive. After further evaluation, no cause of acute pancreatitis was found (ultrasonography, serum calcium, parathyroid hormone, and serum triglycerides were normal).

During his hospitalization, approximately 12 days after jaundice onset, the patient developed right-side 7th cranial nerve palsy (lower motor neuron type) (Figures 1C, 1D). On etiological workup for Bell’s palsy, viral etiology such as herpes simplex virus (HSV) 1 and 2, cytomegalovirus, varicella-zoster virus, and Epstein-Barr virus (EBV) were negative based on polymerase chain reaction (PCR) findings. CECT temporoparietal bone was also normal. Further workup for other etiologies, such as an anti-nuclear antibody (ANA) by immunofluorescence (IF) and serum angiotensin-converting enzyme (ACE), also showed normal values. The patient was managed with standard treatment guidelines for acute hepatitis and acute pancreatitis. A neurology consultation was conducted for Bell’s palsy, and he was advised to take oral prednisolone for 14 days.

The patient improved symptomatically with treatment. His LFT and facial drooping improved on further follow-up (Table 1).

**Table 1:** Laboratory features of the patient during and after the episode of acute viral hepatitis E

Variables	Normal values	Values on presentation	Follow-up values
Hemoglobin (g/dL)	13–16	11	12.4
Total leukocyte count (per/mm^3^)	4,000–11,000	3,700	4,670
Platelet count (lacs/mm^3^)	1.5–4.5	1.72	2.12
Total bilirubin (mg/dL)	0.6–2	8	2.15
Conjugated bilirubin (mg/dl)	0.2–1.2	4	1.05
Aspartate aminotransferase (U/L)	<40 U/L	302	45
Alanine aminotransferase (U/L)	<40 U/L	355	32
Alkaline phosphatase (U/L)	<150 U/L	971	324
Albumin (g/dL)	3.5–5.5 g/dL	3.2	3.4
Protein (g/dl)	6–8.5 g/dL	6.5	7
Amylase (U/L)	30–110 U/L	490	44
Lipase (U/L)	20–160 U/L	356	67
International normalized ratio (INR)	0.8–1.1	1.2	1.1

## Discussion

Reports of hepatitis E outbreaks were first reported from Kashmir, India by Khuroo et al. However, the isolation of the virus and its characterizations were performed after an outbreak in a Russian military group [8]. It is an RNA virus mainly transmitted through the fecal-oral route [9]. While hepatitic illness is mostly self-limiting, the associated mortality rate is exceedingly higher in pregnant females [1,8]. Atypical extrahepatic manifestation in patients with acute viral hepatitis E is not uncommon [3,4,5]. Our patient had acute pancreatitis and Bell’s palsy.

Our patient presented with typical prodromal features with cholestatic jaundice. Severe epigastric pain pointed towards the diagnosis of pancreatitis. However, gallstone disease, pancreatic head mass, and IgG4-related diseases should also be considered as differential diagnoses in patients presenting with cholestatic jaundice and pancreatitis [10]. A bedside USG would rule out a stone disease in such cases. Acute hepatitis E infection is also associated with various autoimmune phenomena [11,12,13]. Previous reports have shown that hepatitis E may present as cholestatic jaundice in a subset of patients [14]. Pokhrel et al. have reported a case of acute cholestasis in an eight-year-old male with hepatitis E infection [15].

Hepatitis E infection also has been implicated in the pathogenesis of autoimmune hepatitis [11]. While the underlying mechanism for such manifestations is poorly understood, molecular mimicry and CD+ T cell-mediated mechanisms have been often implicated. Though autoimmune cholestatic liver diseases are uncommon in India, acute viral hepatitis E and A should be ruled out in specific contexts [11]. Viruses like mumps and rubella are major infective causes of acute pancreatitis [16]. Hepatitis A infection has also been implicated in the etiology of acute pancreatitis [17]. It often follows an uneventful course and resolves spontaneously.

Bell’s palsy has been associated with various infective etiology. The hepatotropic virus causing Bell’s palsy is relatively rare [6,7,18]. Studies have documented Bell’s palsy with acute viral hepatitis A, B, and C. Kamar et al. reported a variety of neurological manifestations of HEV infection including GBS, encephalitis, and neuritis [18]. Besides Bell’s palsy, other neurological manifestations include GBS, brachial neuritis, pseudotumor cerebri, acute transverse myelitis, and meningoencephalitis. Jha et al. reported a similar case of Bell’s palsy with viral hepatitis infection. However, recent reports have found no association between Hepatitis E infection and Bell’s palsy [6,7]. Our case involves the second report from India regarding this rare association. The exact mechanism for such neurological manifestations is unknown. Molecular mimicry and cross-reactivity with neuronal proteins can explain neurological manifestations like GBS [9,12]. Other infections, including tuberculosis, have been implicated in GBS’s pathogenesis [19,20]. Our patient developed Bell’s palsy after the jaundice set in. The icteric phase resolved spontaneously, and the steroid did not cause any flare of underlying infection in this case up to three months of follow-up.

## Conclusions

Acute viral hepatitis may present with predominant cholestatic hepatitis. Acute pancreatitis and neurological manifestations are major extrahepatic manifestations of acute viral hepatitis and should be ruled out in an appropriate clinical setting.
